# Electroporation- and Liposome-Mediated Co-Transfection of Single and Multiple Plasmids

**DOI:** 10.3390/pharmaceutics17070905

**Published:** 2025-07-12

**Authors:** Uday K. Baliga, Anthony Gurunian, Aitor Nogales, Luis Martinez-Sobrido, David A. Dean

**Affiliations:** 1 Department of Pathology, University of Rochester, Rochester, NY 14620, USA; uday_baliga@urmc.rochester.edu; 2 Department of Pediatrics, University of Rochester, Rochester, NY 14620, USA; agurunia@u.rochester.edu; 3 Center for Animal Health Research, CISA-INIA-CSIC, 28130 Madrid, Spain; nogales.aitor@inia.csic.es; 4 Texas Biomedical Research Institute, San Antonio, TX 78227, USA; lmartinez@txbiomed.org

**Keywords:** transfection, electroporation, lipofection, plasmids, multiple plasmids, gene delivery, gene electrotransfer

## Abstract

**Background/Objectives:** Co-transfection of multiple DNAs is important to many research and therapeutic applications. While the optimization of single plasmid transfection is common, multiple plasmid co-transfection analyses are limited. Here we provide empirical data regarding multiple plasmid co-transfection while altering the number of species of plasmids transfected (up to four different plasmids) and the amount of plasmids/cell using the two most common non-viral techniques, electroporation and lipofection. **Methods:** A549 human lung epithelial cells were transfected using lipofectamine 2000 or electroporation with combinations of plasmids, each expressing one of four different fluorescent proteins from the CAGG promoter. Twenty-four hours later, cells were analyzed by spectral flow cytometry to determine the number of cells expressing each fluorescent protein and the amount of fluorescent signal of each protein in a cell. **Results and Conclusions:** For electroporation, while the fraction of cells expressing plasmids increased with increasing amounts of DNA, increasing the number of plasmid species did not alter the fraction of expressing cells and had no effect on levels of expression in individual cells. By contrast, for lipofection, the fraction of cells expressing plasmids was not affected by the amount of DNA added but both the fraction of cells expressing and the level of protein produced in these cells decreased for each plasmid species as the number of delivered species increased. For both lipofection and electroporation after single plasmid transfection, the expressing cells had greater numbers of plasmid copies/cell than non-expressing cells. Multiple plasmid lipofection resulted in more plasmid copies/cell in co-expressing than non-expressing cells. Multiple plasmid electroporation was the inverse of this with fewer plasmid copies/cell in co-expressing than non-expressing cells.

## 1. Introduction

During transfection, plasmids are delivered in a stochastic, concentration-dependent manner to all cells, regardless of transfection reagent or method [1,2]. While this is straightforward for transfections of single plasmids, it becomes problematic when multiple distinct plasmids need to be delivered to the same cells. Indeed, the number of individual cells in a “transfected” population that receive all component plasmids decreases as more components are delivered. Most methodologies, including the best cationic liposomes, PEI, nanoparticles, CaPO_4_, electroporation, and ultrasound, range in their efficiencies to transfect cells, largely depending on the method and cell type. While transfection efficiencies in “easily transfectable” cultured cell lines can reach up to 90%, most cells, especially primary cells, show much lower transfection, with efficiencies typically in the 20 to 40% range, but with some dropping well below this. How this affects co-transfection of multiple plasmid species at the same time remains understudied, despite the widespread use of co-transfections in the laboratory and commercially.

The delivery of multiple plasmids to a single cell is highly useful to many research approaches, including viral reverse genetics [3,4,5,6,7], induced pluripotent stem cell (iPSC) generation [8,9,10], gene editing, and the evaluation of multiple gene interactions [11,12,13]. Such multiplasmid delivery is used in vaccine generation, the development of cellular and genetic therapies, disease modeling, and understanding biological pathways. Each of these multigene-dependent approaches involves the co-transfection of cells with multiple plasmids and/or other cargo (e.g., mRNA). Transfection is often carried out using non-viral methods, particularly electroporation and lipofection, due to the simplicity of applying an electric field to a solution of cells or adding liposome complexes directly onto cells in culture. However, the question of how these non-viral transfection methods affect the co-transfection of each plasmid in the mixtures has not been well-studied.

Lipofection involves the encapsulation of cargo (typically DNA or RNA) by different lipids by mixing to form a lipoplex [14]. After cargo is encapsulated, the lipoplex is added to the cells in culture, and complexes are endocytosed or merge with the cell membrane to allow for delivery of the cargo into a cell [15,16,17,18]. Endocytosed cargo must further escape the endosome/lysosome for release into the cytoplasm where they then diffuse to reach target molecules, e.g., RISC for siRNA, or nuclear transport complexes for pDNA [1,19,20,21,22]. Nucleic acid–lipid complex formation and delivery are both untargeted (although charge can affect organ and tissue distribution when delivered in vivo), and lipofection has been shown to work in most cell types in vitro. A common unstated assumption is that equal mixtures of multiple different plasmids (e.g., pCMV–luciferase and pCAG-GFP) results in co-transfection equivalent to that seen using a single plasmid for transfection [23,24]. As each lipoplex is likely to encapsulate multiple cargoes and each cell can take up multiple lipoplexes, this assumption is not unwarranted. However, this assumption lacks strong evidence. We sought to determine if this assumption is true as increasing numbers of different cargoes are co-delivered.

Electroporation and other physical methods of transfection do not require any encapsulation of cargo for delivery to cells. Physical methods use external forces such as electric pulses, pressure, plasma, or sound waves to create pores in a cell membrane. Extracellular cargo can then diffuse into the cell prior to membrane recovery and sealing of the pores [25,26,27,28]. Because many lipid formulations can lead to various degrees of inflammation and toxicity to cells, physical methods are often preferred since no exogenous material apart from the cargo is reacted with the cells. Furthermore, electroporation allows for some degree of cell-specific targeting in vivo based on the wave form, field parameters, electrode type, electrode placement, and method of DNA administration [29,30,31]. Electroporation also has proven to be clinically safe for gene transfer, electrochemotherapy, and at much higher field strengths, irreversible electroporation [32,33,34,35,36,37,38,39,40,41]. Such physical methods lack the encapsulation needs of chemical methods, and thus delivery of cargo is likely truly random to any cell that is electroporated; thus, multiple cargo delivery is theoretically the overlap in transfection efficiencies of each single cargo. We seek to clarify the validity of this theory.

We start by examining statistical modeling of multiple plasmid co-transfection. We then show how transfection of a single plasmid or multiple plasmids affects electroporation or Lipofectamine 2000-mediated transfection. Through the use of different plasmid species expressing different fluorescent proteins but on an identical plasmid backbone, we assess the transfection of single plasmids and co-transfection of multiple plasmids delivered simultaneously to A549 lung epithelial adenocarcinoma cells, a model cell system used in cell and molecular studies on the lung that we have shown serves as a representative model cell and is routinely used in our lab for intracellular and intranuclear DNA trafficking studies [42,43,44,45,46]. Using a spectral unmixing flow cytometer and appropriate and matched fluorescence minus one control, individual spectra from four different fluorescent proteins can be ascertained in a single sample to quantify gene delivery and expression. We assess the expression of each plasmid by comparing the mean fluorescence intensity (MFI) of a given fluorophore between single or multiple transfected cells. The plasmid copy number in transfected cells is also quantified and analyzed in single or co-transfected cells. These data give important insights into optimal methods for transfection and co-transfection.

## 2. Material and Methods

### 2.1. Mathematical Models

**Figure 1:**
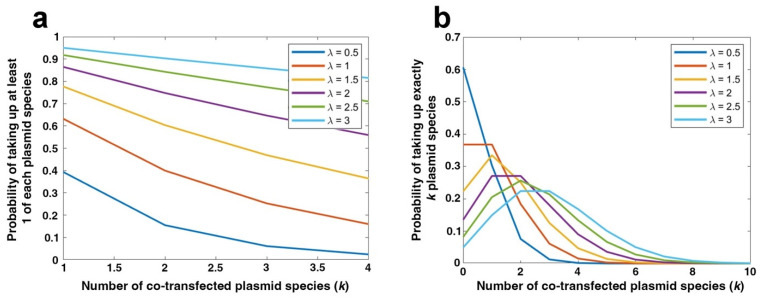
Mathematical modeling of plasmid co-transfection. (**a**) Probability of delivering at least one of each plasmid species (κ) to a cell when multiple plasmids (λ) are delivered. (**b**) A Poisson distribution prediction of transfecting exactly as many plasmids (κ) as one is attempting to deliver (λ). Both plots were generated in Matlab.

Mathematical simulations of transfection (Figure 1) were generated in MATLAB version R2022A (Mathworks, Natick, MA, USA) with the following equations:

(1) $$F\left(k;\lambda \right)\;=\;\frac{\lambda^{k}e^{-\lambda }}{k!}\;$$

(2) $${\left(F_{i\geq 1}\right)}^{n}\;=\;{\left(1\;-\;F\left(0;\lambda \right)\right)}^{n}\;=\;\;{\left(1\;-\;\frac{\lambda^{0}e^{-\lambda }}{0!}\right)}^{n}\;=\;{\left(1\;-\;e^{-\lambda }\right)}^{n}$$

where F(k;λ) is the probability of a cell taking up k plasmid species, λ is the average number of plasmids taken up by a cell, and n is the total copy number of plasmids [47].

### 2.2. Cell Culture

A549 human adenocarcinoma cells (ATCC #CRM-CCL-185, Manassas, VA, USA) were cultured in DMEM supplemented with 10% FBS and 2% antibiotics/antimycotics. All transfections were conducted within the first 20 passages.

### 2.3. Plasmid Preparation

Plasmids expressing different fluorescent proteins were provided by the Martinez-Sobrido Lab. They consist of a pCAGG plasmid backbone each with a different fluorescent protein sequence (eCFP, eGFP, mOrange, and mCherry) inserted at the AvrII site. This results in 5.45 Kb+/−9 bp size plasmids that express a fluorescent protein driven by the hybrid cytomegalovirus (CMV) enhancer and the chicken beta-actin promoter [48]. Plasmids were purified using either Maxiprep or Gigaprep kits (Qiagen, Germantown, MD, USA) per manufacturer recommendations. Plasmid concentration was determined using a Nanodrop One (Thermofisher Scientific, Waltham, MA, USA), and stocks were stored at −80 °C. Working aliquots including multiple plasmid mixtures for co-transfection were diluted to 1 µg/µL in ddH_2_O and stored at −20 °C. Plasmids were confirmed by DNA sequencing with purity and stability assessed by agarose gel electrophoresis.

### 2.4. Electroporation

A549 cells were harvested and resuspended at 5 × 10^6^ cells/mL in electroporation buffer (120 mM KCl, 0.15 mM CaCl_2_, 10 mM K_2_HPO_4_, 10 mM K_2_H_2_PO_4_, 25 mM HEPES, 2 mM EGTA, 5 mM MgCl_2_). Cells (1.25 × 10^6^ in 250 µL) were added to 0.4 cm electroporation cuvettes (Bio-Rad, Hercules, CA, USA) containing the plasmid(s). The amount (µg) and number of plasmid species used varied from 1 to 20 µg/L × 10^6^ cells as noted and between one and four distinct plasmids per transfection (Table 1). Cells were electroporated using a single 20 msec square wave pulse of 300 V, 2000 µF, and 1000 Ohms administered with a Bio-Rad Gene Pulser Xcell System (Bio-Rad, Hercules, CA, USA). Electroporated cells were allowed to recover in complete media for 24 h in a 12-well plate (~5 × 10^5^ cells per well), after which two 50 µL aliquots of media were removed for cytotoxicity measurements followed by harvesting of the cells for FACs analysis and DNA extraction. In all cases, transfections were performed in triplicate, and all experiments were carried out at least three independent times, with representative experiments shown.

### 2.5. Lipofection

Cells were plated in 12-well plates (5 × 10^5^ cells per well) 1–2 days prior to transfection and transfected when 70–90% confluency was reached. Lipofection was carried out using Lipofectamine 2000 (Thermofisher Scientific, Waltham, MA, USA) according to the manufacturer’s instructions. Briefly, a 2:5 ratio of DNA/Lipofectamine was used with Opti-MEM as diluent; the complex was incubated at room temperature for 5 min and then added to wells of cells in a dropwise manner. The plasmid amount varied from 1 to 20 µg for 1 × 10^6^ cells as noted, and lipofectamine was adjusted to the amount of pDNA used. After 4 h, the medium was replaced with DMEM containing 10% FBS and 2% antibiotic/antimycotic solution. Cells were allowed to recover in this complete medium for 24 h, at which point two 50 µL aliquots of media were removed for cytotoxicity measurements followed by harvesting of the cells for FAC analysis and DNA extraction. In all cases, transfections were performed in triplicate, and all experiments were carried out at least three independent times, with representative experiments shown.

### 2.6. Spectral Flow Cytometry

Twenty-four hours after transfection, cells were harvested using trypsin, and half of the cells were fixed in 4% paraformaldehyde and analyzed by FACs within 2 weeks using either a 4-laser (UV-V-B-R) or 5-laser (UV-V-B-YG-R) Cytek Aurora spectral flow cytometer (Cytek Bio, Fremont, CA, USA). Unmixing parameters were determined using single transfection controls and gates based on fluorescence minus one (FMO) controls [49]. FMO controls were the different triple transfection combinations using 2 µg of each plasmid, but all lacking the fourth plasmid in the case of a 4-plasmid transfection; FMO controls for three-plasmid transfection were different two-plasmid transfection combinations using 2 µg, but lacking the third plasmid. Collected data was analyzed using FCS Express (Denovo Software, Pasadena, CA, USA). Total transfection was determined by subtraction of cells negative for all 4 fluorescent proteins (100-Neg%). Representative figures of the gating strategy are in Supplemental Figure S1. All flow cytometry was performed in the University of Rochester Medical Center Flow Cytometry Shared Resource.

### 2.7. Relative Fluorescence Intensity

To observe relative fluorescence intensity, FAC data was used to determine the geometric mean fluorescent intensity (MFI) of cells positive for a given fluorescent protein and then normalized to a control. In single plasmid experiments, the control was the intra-experimental 2 µg sample. In multiple plasmid experiments, each fluorescent protein in a sample was separately normalized to the respective intra-experimental single 2 µg transfection sample. These multiple relative fluorescence values for each different fluorescent protein in a sample were then averaged [49].

### 2.8. Sorting

When the sorting of cells was required, cells were harvested 24 h after transfection using trypsin, placed on ice, and sorted within one to two hours using a 5-laser (UV-V-B-YG-R) Cytek Aurora spectral flow cytometer/sorter. Cells were sorted into singlet cells (total sample), non-expressing, co-expressing (all delivered plasmids), and partial expressing (less than all delivered plasmids) samples. The gating strategy is shown in Supplemental Figure S1.

### 2.9. qPCR for Plasmid Copy Number

DNA was isolated from unfixed, transfected cells using the Qiawave DNeasy kit (Qiagen, Germantown, MD, USA) per manufacturer protocols. Briefly, transfected and control cells were digested and lysed with proteinase K followed by DNA binding to a silica column, washing, and elution (in 200 µL of nuclease-free H_2_O). Aliquots (1 ng/µL) of DNA were made by further dilution in nuclease-free water, and 5 µL of aliquoted DNA was used for each qPCR reaction. Standard curves were made using diluted plasmids (1 ng, 500 pg, 100 pg, 10 pg, 1 pg, 500 fg). A standard curve for cell number was made by extracting DNA from known numbers of cells (6 × 10^5^, 3 × 10^5^, and 1.5 × 10^5^) without input amount standardization. qPCR of DNA from sorted cells had their cell numbers determined by the cytometer, and the input (5 µL) for qPCR was not normalized. Primers to amplify genomic GAPDH and the plasmid ori region (Supplemental Table S1) were used for qPCR with the iQ SYBR Green Supermix (Bio-Rad, Hercules, CA, USA). Ct values above 30 were discarded as either non-specific or due to primer dimer amplification. Using the plasmid mass standard curve, samples were converted to DNA copy numbers based on plasmid size (5.4 kb) and divided by the number of cells calculated by the GAPDH standard curve or sorted population, leading to values for plasmid copies/cell.

### 2.10. Cytotoxicity

Cytotoxicity was measured by lactate dehydrogenase (LDH) release using the Cyquant LDH Cytotoxicity assay (Thermofisher Scientific, Waltham, MA, USA). Media alone was used for background absorbance measurements. Lysed cells were used to calculate 100% cytotoxicity, and untreated cells were used to determine amounts of spontaneous LDH release (0% cytotoxicity).

### 2.11. Statistical Analyses

All gene expression experiments used an n of 3 or greater and were repeated at least 3 times. Sorted qPCR used an n of 3 across two experiments. Error bars are represented as SEM. Statistical significance (*p* < 0.05) was tested using one-way ANOVA followed by a multiple comparisons test using Prism (GraphPad, San Diego, CA, USA).

## 3. Results

### 3.1. Statistical Modeling

Non-viral transfection is often modeled as a random stochastic process such that the delivery of one plasmid is not dependent on or correlated with the delivery of a second or third plasmid [1,2,50,51]. As such, co-transfection efficiency could be calculated by simple multiplication of the transfection efficiency of single plasmids. Two plasmids delivered at 20% transfection efficiency individually would have a 4% co-transfection efficiency with an exponentially decreasing efficiency of co-transfection as the number of distinct plasmids is increased. This would further imply that total transfection would increase to 36% when delivering two plasmids. However, empirically in laboratory use, total transfection is often not significantly changed, and co-transfection efficiency is higher than predicted by simple models. Common theories imply this is due to cellular variation in plasmid uptake and expression [52,53,54,55].

Mathematically, a more accurate model for co-transfection follows a Poisson distribution $F\left(k;\lambda \right)=\frac{\lambda^{\kappa }e^{-\lambda }}{\kappa !}$, where F(k;λ) is the probability of a cell taking up k plasmid species, and λ is the average number of plasmids taken up by a cell (Figure 1a,b) [47]. In this model, two plasmids with different sequences (e.g., a GFP plasmid and an mCherry plasmid) would be considered two separate species. This model can be improved by noting that multiple distinct plasmids are being delivered, leading us to include the probability that each of the distinct plasmids is among those delivered to a given cell. Assuming that plasmid uptake is an independent process (i.e., the uptake of one plasmid has no effect on the uptake of another plasmid), the uptake of each individual plasmid would also follow a Poisson distribution [56]. This implies that the average number of plasmids taken up by a cell is the product of the number of plasmid species and the average total number of plasmids, or nλ. Since the random variables are independent, the probability of taking up at least one copy of each plasmid species is the product of the n individual probabilities, resulting in ${\left(F_{i\geq 1}\right)}^{n}={\left(1-F\left(0;\lambda \right)\right)}^{n}={\left(1-\frac{\lambda^{0}e^{-\lambda }}{0!}\right)}^{n}={\left(1-e^{-\lambda }\right)}^{n}$. This equation is graphically represented in Figure 1b. In essence, as the cell takes up more total plasmids (n), the higher the fraction of cells that will take up more plasmid species and the fewer total plasmids the cells take up, the less likely they are to be transfected with multiple plasmid species. This model has been used to assess the transfection of two plasmid species (n), in which the number of plasmids taken up by a cell (λ) were varied by adjusting the amount of plasmid delivered in µg [47]. The authors using this model predicted that an increase in the amount of plasmids delivered correlated to an increase in co-transfection, theoretically reaching 100% co-transfection with high enough amounts of plasmid, but they provided no empirical confirmation for these predictions.

Achieving less than 100% co-transfection implies that there are cells that are transfected but only with some, but not all, of the added plasmid species. This means that the transfected population of cells is more heterogenous than typically assumed and implies that work reliant on having co-transfection of all added plasmid species without further selection is less convincing. To observe co-transfection heterogeneity, we delivered up to four different plasmids while keeping either the total amount of nucleic acid delivered (λ) constant while altering the number of plasmid species (n) or increasing the amount of nucleic acid delivered (λ) in proportion to the number of plasmid species being delivered. Using this methodology, we developed an evidence-based empirical analysis of multiple plasmid delivery. This allowed us to determine the optimal methods for co-transfection of multiple plasmids and study differences between single plasmid species transfection and multiple plasmid species co-transfection.

### 3.2. Transfection Efficiency Based on Transgene Expression Following Plasmid Delivery

#### 3.2.1. Electroporation of a Single Plasmid

**Figure 2:**
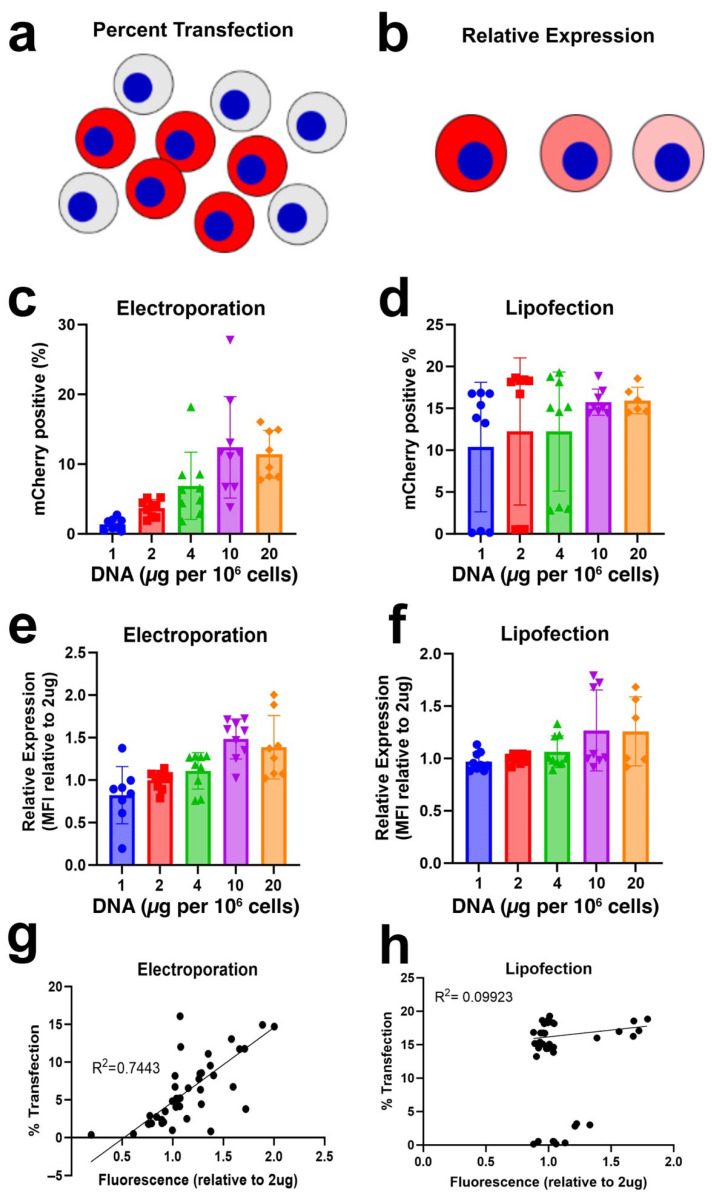
Single transfection of varying DNA amount. (**a**). Graphical representation of the percent of cells expressing mCherry (red) resulting from transfection. (**b**). Graphical representation of relative mCherry expression (red) in individual cells. (**c**). Percent of mCherry-expressing cells 24 h after electroporation as a function of increasing amounts of transfected mCherry plasmid. Cells were electroporated using the same conditions but with increasing amounts of plasmid. (**d**). Percent of mCherry-positive cells 24 h after lipofection with increasing amounts of an mCherry plasmid. (**e**). Relative fluorescent intensity of mCherry-positive cells from C after electroporation. (**f**). Relative fluorescence of mCherry-positive cells from D after lipofection. (**g**). Relationship between the percent of mCherry-positive cells and relative fluorescence of positive cells after electroporation. (**h**). Relationship between the percent of mCherry-positive cells and the relative fluorescence of positive cells after lipofection. The percentage of positive cells was determined by spectral flow cytometry and the fluorescence intensity was measured as the normalized geometric mean fluorescence intensity as described in the Methods Section. In all cases, 8–9 transfections were used for analysis (3 experiments with 3 replicates each). Statistical analysis was by one way ANOVA followed by post hoc Tukey tests for multiple comparisons.

We first quantified total transfection of a single plasmid by transfecting increasing amounts of an mCherry-expressing plasmid, pCAGG-mCherry, into cells. This was achieved by electroporation or lipofection of A549 lung epithelial adenocarcinoma cells using established techniques without optimization. We did not optimize transfection protocols in order to amplify the existing inefficiencies and use roughly the same amount of pDNA/cell for each technique. This allowed us to clearly see differences between experimental conditions that may have been lost if optimized transfection protocols were used that maximized all aspects of the transfection process. We quantified both the percentage of cells that take up and express any plasmid (Figure 2a) and the amount of gene product expressed in a given cell (Figure 2b) 24 h post-transfection. As determined by spectral flow cytometry, electroporation of 2 µg of plasmid/1 × 10^6^ cells resulted in 3.7% transfection on average (i.e., 3.7% of cells showed plasmid expression). Increasing the amounts of pCAGG-mCherry up to 4 µg pDNA/1 × 10^6^ cells increased this average to 6.9% (Figure 2c). When 10 µg of plasmid/1 × 10^6^ cells was used, 12.4% of cells expressed mCherry, but this was not increased when 20 µg pDNA/1 × 10^6^ cells was used for transfection (Figure 2c). Electroporation of increasing amounts of a single plasmid also showed an increase in relative expression (MFI, i.e., the amount of gene product per cell). Almost 1.5 times more mCherry expression per cell was detected using 10 µg pDNA/1 × 10^6^ cells compared to that seen with 2 µg pDNA/1 × 10^6^ cells (Figure 2e). There is a positive correlation (R^2^ = 0.7443) in the relative expression observed and increased amount of plasmid electroporated, although it was not 1:1 (Figure 2g).

#### 3.2.2. Lipofection of a Single Plasmid

We next carried out a similar analysis for cells transfected with Lipofectamine 2000. Lipofection of 2 µg pDNA/1 × 10^6^ cells resulted in 12.4% transfection expression on average. Increasing amounts of plasmid to 4, 10, or 20 µg pDNA/1 × 10^6^ cells increased average transfection up to 15%, although this difference is not statistically significant (Figure 2d). Increasing the amount of plasmid delivered by lipofection only showed a mild increase in MFI or the amount of transgene expression per cell (Figure 2f). Unlike that seen for electroporation, there was no significant correlation between transfection percentage and relative expression (Figure 2h).

#### 3.2.3. Electroporation of Multiple Plasmids

**Figure 3:**
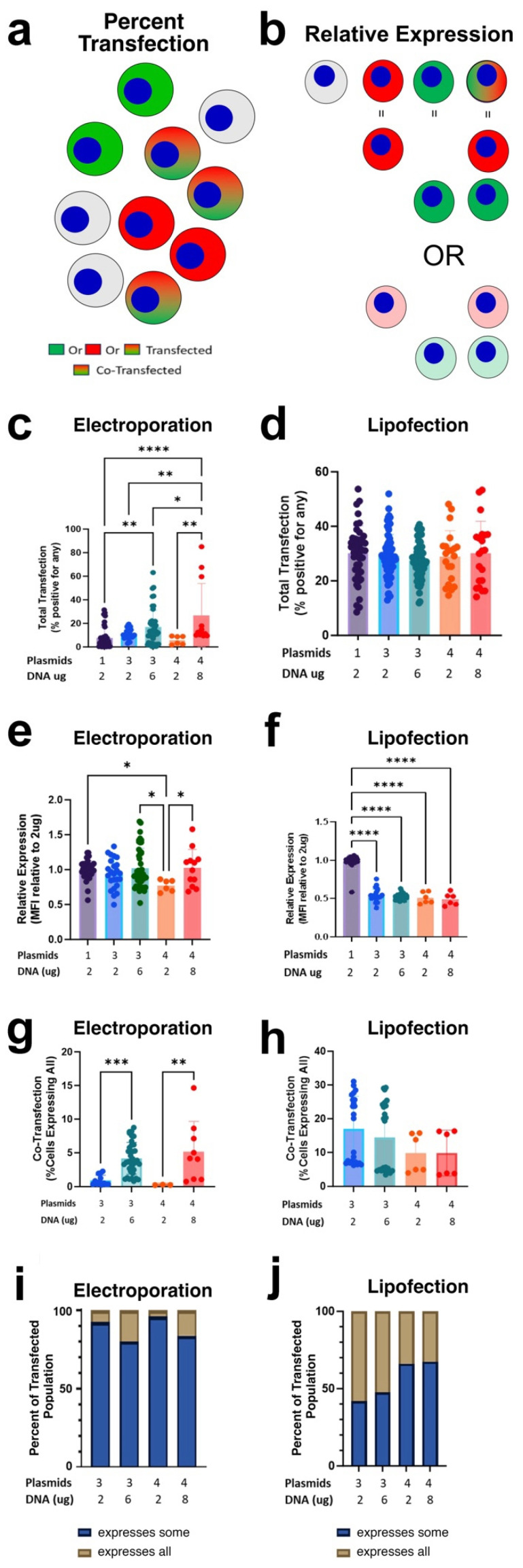
Transfection and relative fluorescent intensity of multiple plasmids. (**a**) Graphical representation of mCherry and GFP co-transfection. Cells transfected with a mixture of pCAGG-mCherry and pCAGG-eGFP can show no expression (non-transfected; gray), GFP or mCherry alone, or both mCherry and GFP. Any cell expressing a fluorescent protein is considered transfected, but only cells expressing both at the same time are considered completely co-transfected. (**b**) Graphical representation of relative mCherry and eGFP expression in individual cells. (**c**) Percent of cells that are expressing any fluorescent protein (total transfection) 24 h after electroporation as a function of increasing amounts of transfected plasmids (single, triple, or quadruple transfection). Cells were electroporated using the same conditions but with increasing numbers of plasmid species (1, 3, or 4) and varying amounts of plasmid (2, 0.67, or 0.5 µg of each plasmid). (**d**) Percent of cells that are expressing any fluorescent protein (total transfection) 24 h after lipofection as a function of increasing amounts of transfected plasmids (single, triple, or quadruple transfection). Cells were transfected with Lipofectamine at a DNA/lipid ratio of 2:5 with increasing numbers of plasmid species (1, 3, or 4) and varying amounts of plasmid (2, 0.67, or 0.5 µg of each plasmid). (**e**) Relative fluorescence intensity of fluorescent-protein-positive cells from (**c**) after electroporation. (**f)** Relative fluorescence intensity of fluorescent-protein-positive cells from (**d**) after lipofection. (**g**) Percent co-transfection in cells after electroporation. The percentage of cells expressing all delivered species in each transfection is shown for 3- and 4-plasmid transfections from (**c**) is shown as a function of total cells present. (**h**) Percent of co-transfection in cells after electroporation. The percentage of cells expressing all delivered species in each transfection is shown for 3- and 4-plasmid transfections from (**d**) is shown as a function of total cells present. (**i**) Percent of co-transfection within the population of transfected cells following electroporation. The percentage of cells expressing all transferred plasmid species as a function of cells that express any or all plasmid species following electroporation-mediated transfection from (**c**) is shown. Partially co-transfected cells (“expresses some”) express fewer than all the transferred plasmid species and completely co-transfected (“expresses all”) express all plasmid species. (**j**) Percent of co-transfection within the population of transfected cells following lipofection. All samples were analyzed via spectral flow cytometry with fresh unmixing controls (single plasmid) and FMOs for each experiment. The percentage of positive cells was determined by spectral flow cytometry and the fluorescence intensity was measured as the normalized geometric mean fluorescence intensity as described in the Methods Section. In all cases, 6–24 transfections were used for analysis (3–8 experiments with 3 replicates each). Statistical analysis was by one way ANOVA followed by post hoc Tukey tests for multiple comparisons. *, *p* < 0.05; **, *p* < 0.01; ***, *p* < 0.001; and ****, *p* < 0.0001.

We next performed co-transfections with combinations of multiple plasmid species to see if the number of plasmid species or the DNA amount itself affected transfection, expression, or co-transfection. The plasmids used expressed either an eCFP, eGFP, mOrange, or mCherry fluorescent protein from the CAG promoter, all on the same pCAGGS plasmid backbone. Transfections were carried out with either a constant or an additive amount of total plasmid to determine whether increasing the mass of pDNA could improve co-transfection. In a constant total co-transfection, each sample had 2 µg of total pDNA/1 × 10^6^ cells; this means that if cells were transfected with one plasmid only, 2 µg of the given plasmid would be used, but if cells were co-transfected with three plasmids, 2 µg/3 or 0.67 µg of each plasmid would be added to the cells, for a total transfection amount of 2 µg of DNA. Thus, each transfection received the same amount of DNA. In theory, a constant amount of pDNA would result in a similar proportion of cells receiving similar amounts of plasmid and thus a similar proportion expressing the plasmids. In an additive total co-transfection, each of the three species of plasmid was present at 2 µg/1 × 10^6^ cells for a total of 6 µg/1 × 10^6^. This was as if each plasmid was used in a single transfection and increased total amount of pDNA (Table 1). It is expected that additive amounts of pDNA would have an additive effect on cells receiving and expressing plasmids. We used spectral flow cytometry to analyze cells expressing at least one of the transfected plasmids included in the total transfection. Only cells expressing all three plasmids in a triple transfection or all four plasmids in quadruple transfection were counted as completely co-transfected (Figure 3a); when fewer fluorescent proteins than the number of plasmids used for the transfection were expressed in a given cell, the cells were counted as partially co-transfected. Relative expression of the fluorescent protein(s) in each cell was also determined (Figure 3b). As expected, electroporation using constant total transfection showed similar total transfection efficiencies (percent of cells expressing transgene) to that of single plasmid transfection (~5–10%), regardless of the plasmid species being delivered. When a constant amount of DNA was used for all transfections (e.g., 2, 0.67/0.67/0.67, or 0.5/0.5/0.5/0.5 µg) and the percentage of cells expressing any or all fluorescent proteins were determined, 7.69 ± 1.38% of cells expressed fluorescent protein when cells were transfected with single plasmids, compared to 11.2 ± 0.93% for cells transfected with three plasmid species, and 5.74 ± 1.47% for cells transfected with four plasmid species (Figure 3c). As expected, additive amounts of pDNA delivered by electroporation in additive co-transfections also increased the total percentage of cells that expressed any or all fluorescent proteins. Cells transfected with 0.67 µg of each plasmid in a triple co-transfection showed expression in 11.2 ± 0.93% of cells compared to 16.8 ± 1.47% when 2 µg of each of the same plasmids were used. Similarly, cells co-transfected with four plasmids expressed in 5.74 ± 1.47% of cells when 0.5 µg of each plasmid was used compared to expression in 26.7 ± 7.86% of cells when 2 µg of each plasmid was used. Stochastic modeling would expect triple transfection to result in ~18.9 to 27% and quadruple transfection to be ~25.24 to 36.28% of cells, which is near the empirical data of additive plasmid delivery. In all cases, increasing the total amount of DNA used for electroporations, either as one plasmid or as multiple plasmids, increased the percentage of cells expressing any or all fluorescent proteins. Surprisingly, the relative expression of each plasmid species in a given cell as determined by MFI was not greatly altered as either the number of plasmids increased or the total amount of DNA used increased (Figure 3e). While a statistically significant drop of MFI was detected in cells transfected with a constant amount of DNA when four plasmids were used in the co-transfection (0.5/0.5/0.5/0.5 µg plasmid), the decrease was less than 20%. When 2 µg of each plasmid was used in the additive co-transfection of four plasmid species, no reduction in MFI was observed (Figure 3e).

We next quantified complete co-transfection, the delivery, and expression of all relevant plasmids within a single cell. We found that when using constant total amounts of pDNA, the efficiency of co-transfection is very low in both triple (0.91 ± 0.21%) and quadruple (0.24 ± 0.02%) transfections. The percentages of co transfected cells were improved when the total amount of input DNA was increased in the additive co-transfections (triple co-transfection showed complete co-transfection in 4.17 ± 0.39% of cells and quadruple additive co-transfection expressed in 5.16 ± 1.50% of cells)(Figure 3g). Stochastic model expectations based off our single plasmid transfection efficiency would predict extremely low amounts of co-transfection in triple (0.0125–0.1%) and quadruple transfection (0.000625–0.01%) which is significantly less than experimentally shown. When normalized to the percentage of cells that expressed a transgene, 7.5% of the triple co-transfected population and 3.96% of the quadruple co-transfected population expressed all delivered plasmids simultaneously in cells transfected with constant total pDNA (Figure 3i). In the case of additive co-transfections, 19.9% of expressing cells transfected with three plasmids expressed all three proteins, and 16.2% of expressing cells transfected with four plasmids expressed all four proteins. Simply expressing one transgene is therefore correlated with the expression of additional transgenes. For electroporation-mediated transfection, as more plasmid species are delivered to cells, the numbers of cells that express any transgene increases, aligning with the random, non-selective delivery model for co-transfection. However, the co-expression of multiple transgenes is greater than expected by the stochastic model.

#### 3.2.4. Lipoplex Transfection of Multiple Plasmids

Total transfection, relative expression, and degree of co-transfection were then analyzed for liposome-meditated transfections. As expected from the single plasmid transfection data, the percentage of cells that expressed any or all delivered transgenes (~30%) did not change as the dose of transfected DNA increased or as the number of plasmid species increased (Figure 3d). Stochastic model predictions of total transfection would be nearly 90% in triple transfection and approach 100% in quadruple transfection, significantly greater than observed. However, we did detect a 50% decrease in the relative expression of each plasmid, as determined by MFI, in all co-transfected cells when compared to cells transfected with a single plasmid. This reduction in MFI is not rescued regardless of plasmids being delivered additively (Figure 3f). When 0.67 µg of each plasmid was used in a triple plasmid co-transfection, the expression of the fluorescent protein decreased by 50%, the same amount of decrease as seen when 2 µg of each of the three plasmids were used in an additive triple plasmid co-transfection. When the percentage of cells that take up and express all plasmid species (complete co-transfection) was determined, we found that as the number of different plasmid species increased, the percent of cells expressing all of the plasmids decreased (Figure 3h). When cells were transfected with a single plasmid, 30.1 ± 1.52% of cells expressed the plasmid. However, when three plasmids were co-transfected into cells, only 16.9 ± 2.03% of cells expressed all plasmids when 0.67 µg of each plasmid was used, and 14.4 ± 2.09% of cells expressed them when 2 µg of each plasmid was delivered. For four plasmid co-transfections, 9.81 ± 2.37% and 9.81 ± 2.77% of cells expressed all four fluorescent proteins when 0.5 µg or 2 µg of each plasmid were used, respectively. Stochastic model expectations would be 2.3–3.2% triple-transfected cells and 0.67–1% quadruple-transfected cells.

The percentage of cells expressing all plasmid species in a co-transfection relative to the total transfected population was not affected by the mass of each DNA added but decreased as the number of plasmid species increased (Figure 3j). Of cells expressing any transgene in a triple transfection, 58.2% and 52.4% of expressing cells were co-transfected, expressing all three fluorescent proteins when 0.67 µg or 2 µg of each plasmid were used, respectively. Of expressing cells in a quadruple transfection, 33.9% and 32.6% of expressing cells were co-transfected, expressing all four fluorescent proteins when 0.5 µg or 2 µg of each plasmid were used in a four-plasmid co-transfection, respectively. Taken together, co-transfection by lipofection is observed but is decreased dependent on the number of different plasmid species, not the amount of added DNA and, unlike electroporation, is not rescued by increasing the amount of plasmid being delivered. These results do not fit the simplified stochastic model; total transfection is not increased as expected by stochastic models, and co-transfection is greater than expected. There does appear to be a correlation between cells transfected with any plasmid and their expression of additional transgenes.

### 3.3. Cytotoxicity

**Figure 4:**
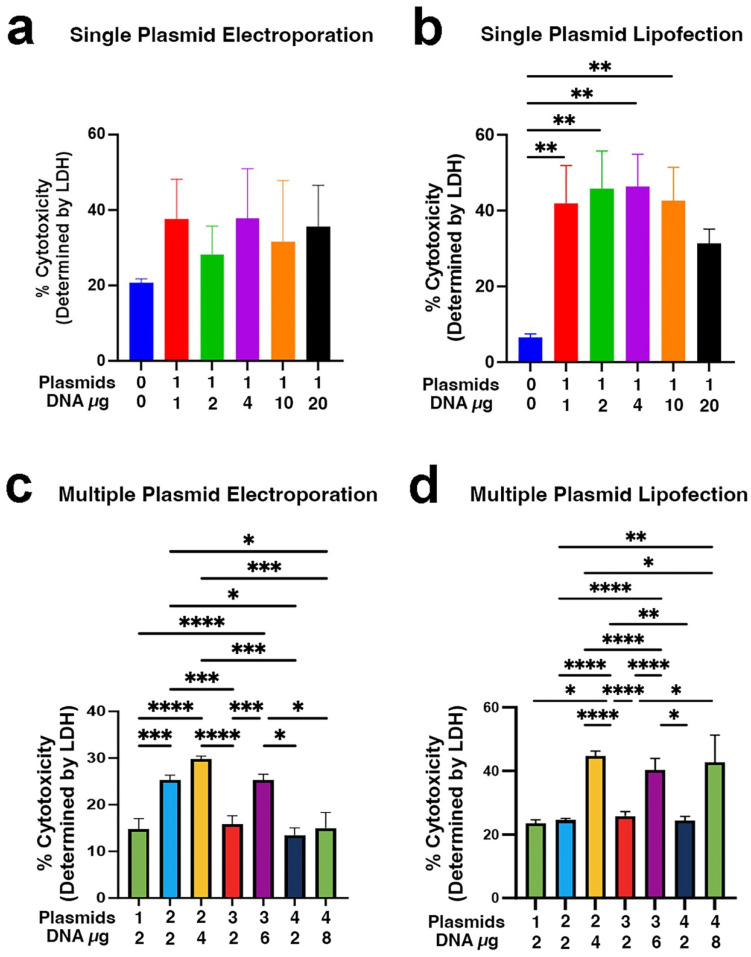
Cytotoxicity. Twenty-four hours after transfection, media was removed from transfected cells and assayed spectrophotometrically for lactate dehydrogenase as a measure of cytotoxicity. Cell lysates also were prepared, and total cellular lactate dehydrogenase was set to 100%. (**a**) Cytotoxicity after electroporation with varying amounts of pCAGG-mCherry. (**b**) Cytotoxicity after lipofection with varying amount of pCAGG-mCherry. (**c**) Cytotoxicity after electroporation with multiple plasmid species and/or increased amount of total DNA. (**d**) Cytotoxicity after lipofection with multiple plasmids and/or increased amount of DNA. In all cases, 6–9 transfections (from Figure 3) were used for analysis (at least 3 experiments with 3 replicates each). Statistical analysis was by one way ANOVA followed by post hoc Tukey tests for multiple comparisons. *, *p* < 0.05; **, *p* < 0.01; ***, *p* < 0.001; and ****, *p* < 0.0001.

A common concern in transfections is that cytotoxicity may increase as the amount delivered increases, likely due to the addition of the bacterial endotoxin present that was not removed during pDNA isolation [57,58,59]. To determine whether increasing the number of plasmid species had any effect on toxicity apart from the levels of pDNA, we gathered media samples 24 h after transfection and used a lactate dehydrogenase (LDH) assay to quantify cell death. Electroporation-induced cytotoxicity was highly variable and unrelated to the amount of plasmid delivered or the number of plasmid species delivered (Figure 4a,c). Consistent with prior work, lipofection toxicity increased as the amount of total pDNA increased (Figure 4b) [58]. Lipofection of equal amounts of total pDNA kept cytotoxicity at approximately 20%, regardless of how many plasmid species were delivered. Thus, for either transfection method, the number of distinct plasmid species used has little if any effect on toxicity.

### 3.4. Plasmid Copy Number

**Figure 5:**
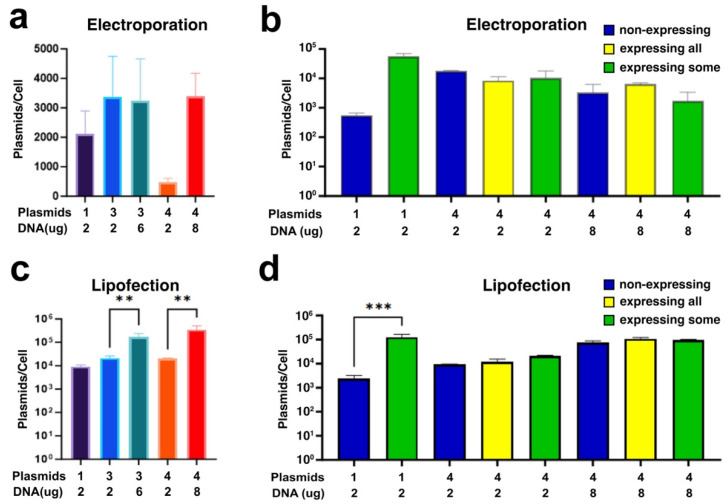
Plasmid copy number. (**a**) Plasmid copy number in electroporated cells as determined by qPCR. DNA was isolated from cells 24 h after electroporation and used for qPCR. A standard curve made with plasmid was used for quantification based on ∆∆Ct analysis. (**b**) Plasmid copy number in electroporated cells as a function of transgene expression; 24 h after electroporation, cells were sorted for fluorescent protein expression into non-expressing (blue), expressing some but not all plasmid species (yellow), or expressing all plasmid species (green) populations. (**c**) Plasmid numbers in Lipofectamine 2000-transfected cells determined by qPCR. DNA was isolated from cells 24 h after electroporation and used for qPCR. A standard curve made with plasmid was used for quantification based on ∆∆Ct analysis. (**d**) Plasmid copy number in lipofected cells as a function of transgene expression; 24 h after electroporation, cells were sorted for fluorescent protein expression into non-expressing (blue), expressing some but not all plasmid species (yellow), or expressing all plasmid species (green) populations. GAPDH gDNA as a standard curve to determine cell number in (**a**,**c**). Cell numbers in (**b**,**d**) were determined during cell sorting. Statistical analysis was by one way ANOVA followed by post hoc Tukey tests for multiple comparisons. **, *p* < 0.01; ***, *p* < 0.001.

We next looked at whether the differences in plasmid co-transfection between lipofection and electroporation were related to the amount of plasmids delivered to the cells. Using qPCR to determine the plasmid copy number, as well as the number of cells, we found that multiple plasmid electroporation resulted in ~2000–3400 copies of pDNA/cell (Figure 5a). Electroporation using 0.5 µg of each of the four plasmids (2 µg total DNA) resulted in a significant reduction in copy number compared to 2 µg of a single plasmid and may partially explain the lower MFI observed in these samples (Figure 3e). Similarly to the relative expression, this copy number reduction for the four-plasmid co-transfection was “rescued” by increasing the amount of total plasmid to 8 µg (Figure 5a). Liposome-mediated transfection resulted in between 7000 and 34,000 copies of pDNA/cell. Unlike the case with electroporation, copy numbers of transfected plasmids increased as the amount of DNA was increased (Figure 5c). Whether using electroporation or lipofection, the simplified Poisson distribution models of the plasmid copy numbers observed would predict nearly all cells to be transfected, as well as co-transfected. However, our previous data indicates this is not true.

We then assessed whether the plasmid number correlated to the co-expression of the plasmids. Cells transfected as above were sorted into populations that expressed no plasmids, expressed some of the delivered plasmid species, or expressed all of the delivered plasmid species (Figure 5b,d). In both Lipofectamine and electroporation transfected cells, non-expressing cells had reasonably high copy numbers of the plasmid despite showing no transgene expression. Lipofection gave 2331 to 74,750 plasmid copies/non-expressing cell, while electroporation gave between 552 and 17,935 plasmid copies/non expressing cell. Again, Poisson distribution models would expect these cells that receive even 500 plasmids to be successfully co-transfected, and they would be above the “minimum threshold” for expression indicated by others [52]. In transfections of single plasmids, the positively expressing population had a much greater number of plasmid copies/cell compared to the non-expressing cells with 125,000 plasmid copies/cell for Lipofectamine-transfected cells and ~56,000 plasmid copies/cell for electroporated cells (Figure 5b). In multiple plasmid transfection, however, when multiple plasmids were delivered to cells using electroporation or lipofection, there was no statistical difference in plasmid numbers between non-expressing or transgene-expressing cells for either technique, although the copy number for liposome-transfected cells did increase slightly, but not with statistical significance, in expressing cells as the mass of transfected DNA increased (Figure 5b,d). These data indicate that a plasmid “minimum” is not a sole necessity for expression. Furthermore, while increased plasmid copy numbers are correlated with single transgene expression, this does not apply to multiple transgene expression.

## 4. Discussion

It is often assumed that transfection of multiple plasmid species is straightforward and almost always results in effective gene delivery of all desired plasmid species [60,61]. As a result of this assumption, co-transfection with multiple plasmids has received surprisingly little experimental attention, despite the widespread use of co-delivered reporter plasmids to indicate delivery or efficiency of a plasmid expressing a gene of interest [60,62,63]. We have shown that when using electroporation, the total transfection efficiency is improved by increasing the mass amount of a single plasmid, as expected. Co-transfection of multiple plasmid species is also improved when the amount of each pDNA cargo is increased. Furthermore, when using electroporation to deliver multiple plasmids, the relative expression of each plasmid within a transfected cell remains very close to the levels seen following transfection of a single plasmid. By contrast, when using lipofection with Lipofectamine 2000, there is little dependency on the amount of plasmid used; the total number of transfected cells is more constant, regardless of the amount of plasmid used for transfection or the number of plasmid species being delivered. Surprisingly, the expression in any given cell (relative MFI) of multiple plasmid species when delivered by lipofection is cut in half relative to single transfection controls, despite generally better co-transfection than electroporation. This average reduction is not relative to the number of additional plasmids delivered. Furthermore, when using lipofection, complete co-transfection (expression of all plasmid species) is decreased and is dependent on the number of plasmid species being delivered, which cannot be rescued by increasing the amount of pDNA in the transfection. Lipofection may thus fail to generate appreciable co-transfection when many (>4) different plasmid species are used. Each additional plasmid species further reduces the number of cells receiving all plasmids and it is highly likely that this trend would continue for additional plasmid species. In cells that are hard to transfect, such as primary fibroblasts, a generally low transfection efficiency may further reduce the number of co-transfected cells.

The two primary models proposed to describe co-transfection efficiency yield very different predictions for the fraction of productively transfected cells that take up and express all plasmid species that are present in the transfection mixture. In the first model, co-transfection is the product of random, independent uptake of each individual plasmid species. This predicts that when 50% of the cells are transfected, the fraction of productively transfected cells expected to take up and express four different plasmid species is roughly 6%. Thus, unless close to 100% of the cells are transfected, the fraction of cells expressing four different plasmids would be minor. Based on our unoptimized transfection efficiencies of single plasmids here that gave between 4% and 15% of total cells expressing transfected plasmids (Figure 2), between 0.01% and 0.34% of the transfected cells would be expected to express all four different species, values that are much lower than that observed for either transfection method. By contrast, in the binomial expansion model, if the number of cells that take up DNA is much greater than the number of different plasmid species, there is a high likelihood that all cells will take up at least one of each of the different species. For instance, if 10 plasmids are taken up by a given cell and there are 4 different plasmid species, according to the Poisson model, greater than 90% of the transfected cells will take up at least one of each of the 4 plasmid species. Our data on DNA uptake by liposome-transfected or electroporated cells (either expressing or not) shows that more than 2000 plasmids are detected per cell, well above the 10 plasmids used in this example, suggesting that almost all transfected cells should express all 4 plasmid species based on this model. Yet, as the data shows, the fraction of productively transfected cells that express all four plasmids is between 20 and 30%, not 100%. Thus, our data show that neither model fully describes co-transfection; co-transfection is neither as dire as predicted by stochastic models nor as good as that predicted in the Poisson distribution model, but rather lies in between, with room for control and improvement.

The electroporation and lipofection methods used in this study were not optimized in order to amplify the inefficiencies observed. It is likely that parameter optimization, such as increased number of electric pulses, field strength, pulse length, or different lipid/plasmid ratios could improve both total transfection and co-transfection. However, these parameters cannot address the underlying stochastic nature of pDNA delivery to a given cell, the intracellular trafficking of the delivered pDNA, or the ultimate pDNA expression. Another limitation of our study is the use of a single cell line (A549). It is possible and likely that slightly different results could be obtained in other cell lines more susceptible to electroporation and/or liposome-mediated transfection. Plasmid size, backbone, and the inserted gene(s) were kept consistent in this work but are also possible variables that may affect co-transfection and relative expression. Differing plasmid sizes or backbones may have different delivery efficiency or be expressed differently, and different genes can have varied regulation, all of which may impact co-transfection as well [14,57,64,65]. Finally, we have used only one liposomal transfection reagent, Lipofectamine 2000, for lipofection studies, and it is possible that different lipid formulations may show differing outcomes. However, since all liposome transfection reagents form complexes with DNA by mixing, we would expect that differences in the DNA delivery of multiple plasmid species would be minor and more due to the methods used for mixing and complex formation as opposed to uptake of the final complexes, which has been shown to be relatively similar for multiple lipid formulations.

Previous studies to determine the copy number of plasmids in transfected cells have shown that the vast majority of cells present during the transfection do indeed take up a significant number of plasmids, regardless of the method of transfection [53,66,67,68]. Tseng et al. found that >95% of cells exposed to liposome–DNA complexes took up >20,000 copies of DNA per cell when an over 100-fold range of DNA concentrations were added to the cells [66]. When the copy number of DNA was correlated with levels of gene expression, cells with fewer than 20,000 plasmids showed very low transgene expression, but when >10^6^ DNA copies per cell were detected, essentially 100% of the cells showed strong gene expression. While looking at the copy number of plasmids in the nucleus following PEI or liposomal transfections, Szoka and colleagues showed in A549 cells that between 75,000 and 50,000 plasmids per nucleus were detected and that this represented about 10 to 25% of the total intracellular DNA [68]. Moreover, when cells were sorted for transgene expression, cells expressing low levels of transgene displayed <1000 copies of DNA per nucleus while those expressing high transgene levels had three-fold higher plasmid copies per nucleus [68]. Although we did not measure the levels of plasmid in the nucleus, these numbers reflect the same trends that we observed, at least when single plasmid species were used. However, our finding is that as the number of plasmid species increases, the direct relationship between plasmid copy number and expression may not hold true, which appears unique to the co-transfection of multiple plasmid species.

Our results show that transfection of single plasmids resulted in roughly 2000 to 20,000 DNA copies per cell when transfected by electroporation or lipofection, respectively. However, when transfected cells were sorted into non-expressing and expressing populations, we detected greater numbers of plasmids per expressing cell (60,000 and 125,000 for electroporation or lipofection, respectively). Our numbers are in the range of those reported previously for single plasmid transfections [53,66,67,68]. Following both methods of transfection, we found that even cells that failed to express detectable gene products still showed significant levels of plasmid uptake (552 to 17,935 plasmid copies per cell following electroporation and 2331 to 74,750 plasmid copies following lipofection), but cells that had detectable gene expression contained greater numbers of plasmid per cell (56,000 plasmid copies per cell following electroporation and 125,000 plasmid copies following lipofection). When multiple plasmid species were transfected into the cells, there was less increase in the copy numbers seen in co-expressing cells compared to non-expressing or partial co-expressing cells, and the overall copy numbers were slightly less than those seen for single plasmid transfections. It was only when increasing amounts of DNA were used for transfections that the copy number increased. Interestingly, the increase in plasmid copy/cell delivered by lipofection did not appear to correlate well with plasmid expression or co-transfection. The use of more stringent methods to remove non-nuclear plasmid DNA may help clarify the disconnect between delivery and expression [53].

The conclusions from our electroporation experiments may extrapolate to other physical methods, such as hydroporation or sonoporation since all deliver cargo via similar non-selective transient membrane pores. Using a greater amount of plasmid with these physical methods should improve co-transfection and it is expected that expression of each plasmid species within the transfected cells would be roughly equivalent to that seen in cells following transfection with a single plasmid. The results seen using lipofection may also extrapolate to other non-selective chemical methods that involve endocytic internalization and “escape”. Biomanufacturing, for example, often uses PEI due to its cost and high activity for HEK293T and CHO transfection [69,70]. While both HEK293T and CHO are “easy to transfect”, often reaching single transfection efficiencies of 70–90%, the reduction in co-transfection may still result in a significant loss of transgene production. The reduced co-transfection of multiple plasmid species delivered by lipofection and lower expression of each plasmid species relative to a single plasmid control should be investigated and noted when multiple plasmid lipofection is performed. Either electroporation or lipofection of multiple plasmids may benefit from concurrent multiple selection factors. Whether more selective biochemical transfection methods such as targeted lipid nanoparticles, exosomes, and synthetic nanoparticles, which have a built-in capacity for endosomal/lysosomal escape, also share these co-transfection inefficiencies remains to be tested.

## 5. Conclusions

We have shown that when electroporation is used to deliver a single plasmid species to A549 human adenocarcinoma cells for transfection, the percentage of cells expressing the encoded transgene and the amount of transgene produced per cell is dose-dependent. By contrast, cells transfected using lipofection show very little, if any, difference in transgene expression (both percent of cells transfected and amount of transgene per cell) dependent on the DNA dose used for transfection. Electroporation of multiple plasmid species (three or four) into cells by electroporation showed no difference in the percentages of transfected cells or the amount of expression in a given cell compared to single-plasmid transfected cells at the same DNA dose, but as more plasmid species were delivered to cells, the numbers of cells that expressed all of them decreased, aligning with a random, non-selective delivery model for co-transfection. By contrast, liposomal transfection of cells showed no dose-dependency of the percentage of cells transfected or the amount of transgene per cell when single plasmid species were used. When more than one plasmid species was used for liposomal co-transfection, the amount of transgene expressed per cell decreased as the number of transferred plasmid species increased, and the percentage of cells expressing all co-transfected plasmids decreased as the numbers of plasmid species increased. Copy numbers of transfected plasmids differed for transfection by the two techniques, with electroporation giving 2000–4000 plasmid copies/cell and lipofection giving 7000–30000 plasmids per cell. Plasmid copy numbers were not altered by increasing the number of plasmid species used but could be increased by the amount of plasmid used. In both transfection methods, cells that failed to express any transgene contained high levels of plasmids but those that expressed transgene had higher copy numbers of intracellular pDNA. However, higher plasmid copy numbers did not further correlate with transgene co-expression. Further work on the molecular mechanisms specific to multiple plasmid co-transfection are warranted due to the potential cost benefits and avoidance of experimental artifacts.

## Figures and Tables

**Table 1:** Transfection strategy.

Sample	Plasmid				
	eCFP	eGFP	mOrange	mCherry	Total DNA
CFP only	2 µg	-	-	-	2 µg
GFP only	-	2 µg	-	-	2 µg
mOrange only	-	-	2 µg	-	2 µg
mCherry only	-	-	-	2 µg	2 µg
Triple transfection (constant mass)	0.67 µg	0.67 µg	0.67 µg	-	2 µg
Triple transfection (additive)	2 µg	2 µg	2 µg	-	6 µg
Quadruple transfection (constant mass)	0.5 µg	0.5 µg	0.5 µg	0.5 µg	2 µg
Quadruple transfection (additive)	2 µg	2 µg	2 µg	2 µg	8 µg

## Data Availability

Data is provided within the manuscript or Supplementary Information Files. Additional data is available from David A. Dean on request.

## Supplementary Materials

The following supporting information can be downloaded at https://www.mdpi.com/article/10.3390/pharmaceutics17070905/s1, Figure S1: Gating strategy for cell sorting. Multiplasmid Gating scheme in a 4 plasmid species transfected sample. Cells are gated on Forward Scatter Area (FSCA) and Side Scatter Area (SSC-A)and then single cells are confirmed by Forward Scatter Height (FSC-H) and FSC-A. Following this, CFP and GFP are separately gated into “2 negative” or double positive populations. The 2 negative population is further gated on mOrange and mCherry showing a population fully negative for all fluorescent proteins, all other cells express at least one fluorescent protein. 2 negative gating also shows cells that only express one or two fluorescent proteins. The double positive population is also gated on mOrange and mCherry showing populations that express all 4 fluorescent proteins as well as populations that only express 3. Table S1: Primers for qPCR.
