# Supplementary material for: Electroporation- and Liposome-Mediated Co-Transfection of Single and Multiple Plasmids
Source: Pharmaceutics. 2025 Jul 12;17(7):905. doi: 10.3390/pharmaceutics17070905 (PMC12297979; doi:10.3390/pharmaceutics17070905)
Supplement: Supplementary file 1 [file pharmaceutics-17-00905-s001.zip › pharmaceutics-3733464-supplementary.pdf]

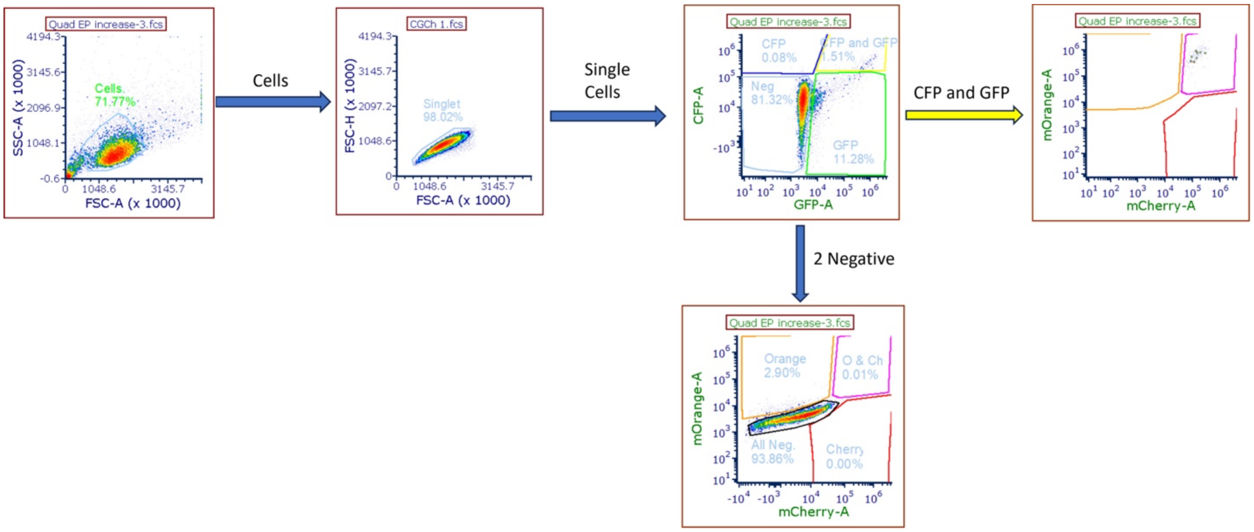

Supplemental Figure S1. Gating strategy for cell sorting. Multiplasmid Gating scheme in a 4 plasmid species transfected sample. Cells are gated on Forward Scatter Area (FSC-A) and Side Scatter Area (SSC-A)and then single cells are confirmed by Forward Scatter Height (FSC-H) and FSC-A. Following this, CFP and GFP are separately gated into “2 negative” or double positive populations. The 2 negative population is further gated on mOrange and mCherry showing a population fully negative for all fluorescent proteins, all other cells express at least one fluorescent protein. 2 negative gating also shows cells that only express one or two fluorescent proteins. The double positive population is also gated on mOrange and mCherry showing populations that express all 4 fluorescent proteins as well as populations that only express 3.

Supplemental Table S1. Primers for qPCR

Primer	Sequence
**GAPDH Forward**	GTCTCCTCTGACTTCAACAGCG
**GAPDH Reverse**	ACCACCCTGTTGCTGTAGCCAA
**f1 Ori Forward**	ATACCTGTCCGCCTTTCTCC
**f1 Ori Reverse**	GAACGACCTACACCGAACTGAG
